# Antibody opsonization enhances MAIT cell responsiveness to bacteria via a TNF‐dependent mechanism

**DOI:** 10.1111/imcb.12239

**Published:** 2019-02-25

**Authors:** Zoltán Bánki, Lisette Krabbendam, Dominik Klaver, Tianqi Leng, Simon Kruis, Hema Mehta, Brigitte Müllauer, Dorothea Orth‐Höller, Heribert Stoiber, Christian B Willberg, Paul Klenerman

**Affiliations:** ^1^ Division of Virology Medical University of Innsbruck Innsbruck Austria; ^2^ Peter Medawar Building for Pathogen Research University of Oxford Oxford UK; ^3^ Division of Hygiene and Medical Microbiology Medical University of Innsbruck Innsbruck Austria; ^4^ Present address: Department of Experimental Immunology Academic Medical Center University of Amsterdam Amsterdam The Netherlands; ^5^ Present address: Department of Dermatology and Venerology Medical University of Innsbruck Innsbruck Austria

**Keywords:** Bacteria, *E*. *coli*, IgG‐opsonization, innate T cells, MAIT cells, TNF

## Abstract

Mucosal‐associated invariant T (MAIT) cells are an abundant human T‐cell subset with antimicrobial properties. They can respond to bacteria presented via antigen‐presenting cells (APCs) such as macrophages, which present bacterially derived ligands from the riboflavin synthesis pathway on MR1. Moreover, MAIT cells are also highly responsive to cytokines which enhance and even substitute for T‐cell receptor‐mediated signaling. The mechanisms leading to an efficient presentation of bacteria to MAIT cells by APCs have not been fully elucidated. Here, we showed that the monocytic cell line THP‐1 and B cells activated MAIT cells differentially in response to *Escherichia coli*. THP‐1 cells were generally more potent in inducing IFNγ and IFNγ/TNF production by MAIT cells. Furthermore, THP‐1, but not B, cells produced TNF upon bacterial stimulation, which in turn supported IFNγ production by MAIT cells. Finally, we addressed the role of antibody‐dependent opsonization of bacteria in the activation of MAIT cells using *in vitro* models. We found that opsonization had a substantial impact on downstream MAIT cell activation by monocytes. This was associated with enhanced activation of monocytes and increased TNF release. Importantly, this TNF acted in concert with other cytokines to drive MAIT cell activation. These data indicate both a significant interaction between adaptive and innate immunity in the response to bacteria, and an important role for TNF in MAIT cell triggering.

## Introduction

Mucosal‐associated invariant T (MAIT) cells represent the most abundant innate‐like T lymphocyte population within the human body comprising up to ~5% of total T cells.^1^ MAIT cells express a semi‐invariant T‐cell receptor (TCR; Vα7.2‐Jα33/12/20 in humans) recognizing intermediates of bacterial riboflavin synthesis pathway if presented on the evolutionarily conserved, nonpolymorphic MHC‐related protein 1 (MR1) by APC.^2^, ^3^ MAIT cells activated in an MR1‐dependent manner may directly kill bacteria‐infected cells by producing granzymes and perforin^4^, ^5^ or augment antibacterial immune mechanisms by secreting proinflammatory cytokines, such as TNF, IFNγ and IL‐17.^6^, ^7^ Results from several studies provide evidence that MAIT cells are critically involved in antibacterial defense against a range of riboflavin‐metabolizing bacteria including *Escherichia coli*,* Pseudomonas aeruginosa, Klebsiella pneumoniae*,* Staphylococcus aureus*,* Staphylococcus epidermidis* and *Mycobacteria*.^6^, ^8^, ^9^, ^10^

Human MAIT cells are characterized by an effector memory phenotype (CCR7^neg^CD62L^lo^CD45RO^pos^CD45RA^neg^CD95^hi^) suggesting an early detection of pathogens and a quick response to bacterial stimulation.^7^, ^11^ Early stages of bacterial recognition by MAIT cells are dominated by the MR1‐dependent augmentation of TCR signaling, which leads to activation and IFNγ production by MAIT cells.^6^, ^8^ MAIT cells react to MR1‐expressing APCs, for example, dendritic cells, B cells and monocytes infected or co‐cultured with bacteria as well as bacterially infected epithelial cells.^4^, ^6^, ^8^ The importance of MR1 in MAIT cell activation and in early control of certain pathogens is emphasized by the increased susceptibility to *K. pneumoniae* and *Francisella tularensis* infections in MR1‐deficient mice.^9^, ^12^

MAIT cells have also been shown to produce IFNγ in response to proinflammatory cytokine combinations (e.g. IL‐12 and IL‐18) independent of MR1.^13^ IL‐12 and IL‐18 are produced by monocytes, macrophages and dendritic cells upon microbial stimuli.^13^, ^14^, ^15^ Previously it was shown that early activation (5 h) of MAIT cells by *E. coli*‐infected APCs is exclusively dependent on MR1, whereas longer incubation (20 h) leads to an additional response of MAIT cells to IL‐12 and IL‐18.^13^ In contrast, the activation of MAIT cells by *E. faecalis* lacking the riboflavin synthetic pathway is solely dependent on IL‐12/IL‐18 production by APCs.^13^ Toll‐like receptor (TLR) stimulation on APCs has been shown to induce IL‐12 and IL‐18 production^15^, ^16^ and particularly the TLR4 ligand LPS has been demonstrated to stimulate IFNγ secretion of MAIT cells in an IL‐12/IL‐18‐dependent manner.^13^ Furthermore, an MR1‐independent IL‐12/IL‐18‐mediated induction of MAIT cell activation by a TLR8 agonist was found, suggesting an additional role of MAIT cells in antiviral responses, which has been confirmed in dengue, hepatitis C and influenza infections.^13^, ^17^

Taken together, bacteria have been demonstrated to induce MAIT cell activation by APCs in an MR1‐ and/or cytokine‐dependent manner. Here, we further investigated MAIT cell activation by different APCs ‐ THP‐1 cells and B cells. Furthermore, we adapted the experimental setting to a more physiological situation in the presence of human serum, in which bacteria can interact with pathogen‐specific IgG antibodies, resulting in immune‐complex formation and complement activation. Thus, bacteria become opsonized with both IgG and complement. We hypothesized that opsonization with IgG and complement impacts the interaction of bacteria with APCs through respective Fcγ‐receptors (FcγR) and complement receptors potentially resulting in a modulation of APC functions. This, in turn, could have an impact on MAIT cell activation. Indeed, here we demonstrate an IgG‐mediated enhancement of MAIT cell activation by macrophages. IgG‐opsonization of bacteria triggers TNF production of macrophages, which in concert with MR1 and other cytokines like IL‐12 and IL‐18 drives MAIT cell activation.

## Results

### THP‐1 cells and BCLs induce different cytokine profiles in MAIT cells in response to *E. coli*

**Figure 1:**
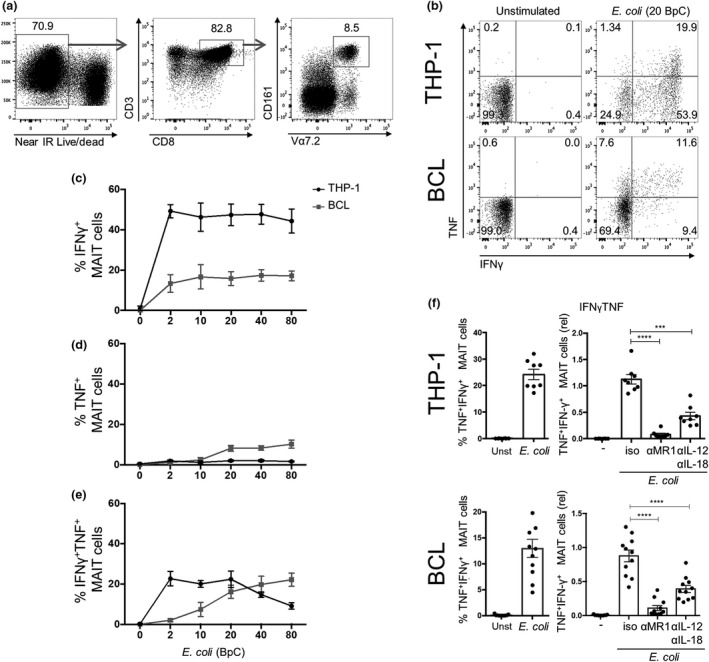
THP‐1 cells and BCLs induce distinct cytokine pattern in human MAIT cells in response to *Escherichia coli*. **(a)** Gating strategy for live, TCRVα7.2^+^ CD161^++^ MAIT cells in CD8 T cells enriched from human PBMCs. Enriched CD8 T cells were co‐cultured for 20 h with THP‐1 or BCLs in the absence or presence of 20 BpC *E. coli*. MAIT cells were analyzed for the expression of IFNγ and TNF by intracellular cytokine staining. **(b)** A representative dot plot for both THP‐1 and BCL co‐cultures is shown indicating percent cytokine positive cells in quadrants. Mean ± s.e.m. of IFNγ^+^ **(c)**, TNF ^+^ **(d)** and IFNγ^+^ TNF ^+^ **(e)** MAIT cells in co‐cultures of enriched CD8 T cells with THP‐1 cells or BCLs in the presence of different BpC *E. coli* is derived from eight donors pooled from two independent experiments. Blocking MR1 and IL‐12/IL‐18 signaling can diminish IFNγ^+^ TNF ^+^ **(f)** MAIT cells in co‐cultures of CD8 T cells with THP‐1 cells or BCLs in the presence of 20 BpC *E. coli* using isotype antibodies (iso) or blocking antibodies for MR1 (αMR1) or IL‐12/IL‐18 (αIL‐12/αIL‐18). Graphs on the left shows the absolute percentage and, on the right, the relative percentage IFNγ^+^ TNF ^+^ MAIT cells.

Previously it was shown that MAIT cells readily respond to bacterial‐infected APCs, like monocytes and B cells.^13^, ^18^ Thus, we compared the ability of THP‐1 cells and B cells (BCL) to activate MAIT cells. We co‐cultured CD8 enriched peripheral blood mononuclear cells (PBMCs) with THP‐1 cells or BCLs in the absence or presence of paraformaldehyde‐fixed *E. coli* for 20 h and analyzed Vα7.2^+^CD161^++^ MAIT cells within the live CD3^+^CD8^+^ cell population (Figure 1a) for the expression of IFNγ and TNF. Different MAIT cell responses were detected by their cytokine expression pattern: IFNγ single positive (IFNγ^+^ MAIT cells), TNF single positive (TNF^+^ MAIT cells) or IFNγ/TNF double positive (IFNγ^+^TNF^+^) MAIT cells. As expected, neither THP‐1 cells nor BCLs could induce a MAIT cell response in the absence of *E. coli* (Figure 1b, unstimulated). When CD8 T cells were co‐cultured with THP‐1 cells together with 20 bacteria per cell (BpC) *E. coli*, we observed IFNγ expression in about 50% of the MAIT cells, which remained stable with increasing BpC (Figure 1c). In contrast, BCLs co‐cultured with CD8 T cells and 20 BpC *E. coli* resulted in less than 20% IFNγ^+^ MAIT cells (Figure 1b, c). Interestingly, BCLs, cultured with high bacterial loads (>10 BpC), induced TNF in about 10% of the MAIT cells, whereas THP‐1 cells induced only a minor fraction of TNF^+^ MAIT cells regardless of the bacterial load (Figure 1b, d). The appearance of IFNγ^+^TNF^+^ MAIT cells strongly depended on the number of BpC added to the cultures. In the BCL culture, increasing BpC correlated with increased IFNγ^+^TNF^+^ MAIT cells, whereas in the THP‐1 cell culture the frequency of IFNγ^+^TNF^+^ MAIT cells decreased at higher bacterial load (Figure 1e). In summary, we found that THP‐1 cells are more potent in inducing IFNγ expression in MAIT cells upon *E. coli* exposure, whereas BCLs are relatively more potent in inducing TNF expression.

MAIT cells respond to bacterial infection via the presentation of riboflavin metabolites by MR1. However, MAIT cells readily respond to IL‐12 and IL‐18, expressed by APCs upon bacterial exposure, by producing high levels of IFNγ.^13^ Therefore, we next compared the contribution of the MR1 pathway and IL‐12/IL‐18 signaling to the activation of MAIT cells by THP‐1 cells and BCLs. The appearance of maximally activated IFNγ^+^TNF^+^ MAIT cells was nearly completely abrogated by blocking the MR1 pathway and significantly reduced by blocking IL‐12/IL‐18 signaling in both co‐cultures (Figure 1f). Similar results were found for IFNγ^+^ MAIT cells (data not shown).

Taken together, the activation of MAIT cells was dependent on both MR1 and IL‐12/IL‐18 signaling pathways, independent of the APC tested with a major role for MR1 signals in triggering the most highly activated (TNF^+^IFNγ^+^) responding MAIT cells, most readily activated by THP1 cells.

### TNF is expressed by THP‐1 cells, but not BCLs upon bacterial stimulation and triggers IFNγ expression in MAIT cells

**Figure 2:**
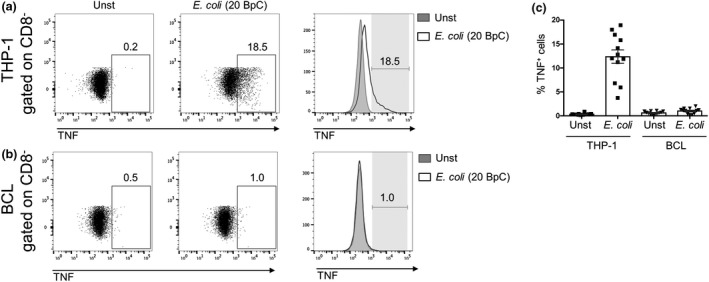
THP‐1 cells but not B cells produce TNF upon bacterial stimulation. THP‐1 cells **(a)** or BCLs **(b)** were co‐cultured with enriched CD8 T cells for 20 h in the absence (Unst) or presence of 20 BpC *E. coli*. THP‐1 cells or BCLs defined by gating on CD8 negative cells were analyzed for TNF expression after intracellular cytokine staining. Numbers next to gates represent percentage of positive cells. **(c)** Mean ± s.e.m. TNF ^+^ THP‐1 or BCLs are shown from 12 donors pooled from three independent experiments.

Since TNF is induced upon monocyte activation,^19^ and since THP‐1 cells provided the strongest stimulation, we explored the expression of TNF in THP‐1 cells and BCLs in the CD8 T cell co‐cultures. TNF expression was observed in 4‐20% of the THP‐1 cells upon exposure to *E. coli* (Figure 2a, c), whereas TNF expression was detected in only a small fraction of the BCLs (Figure 2b, c).

**Figure 3:**
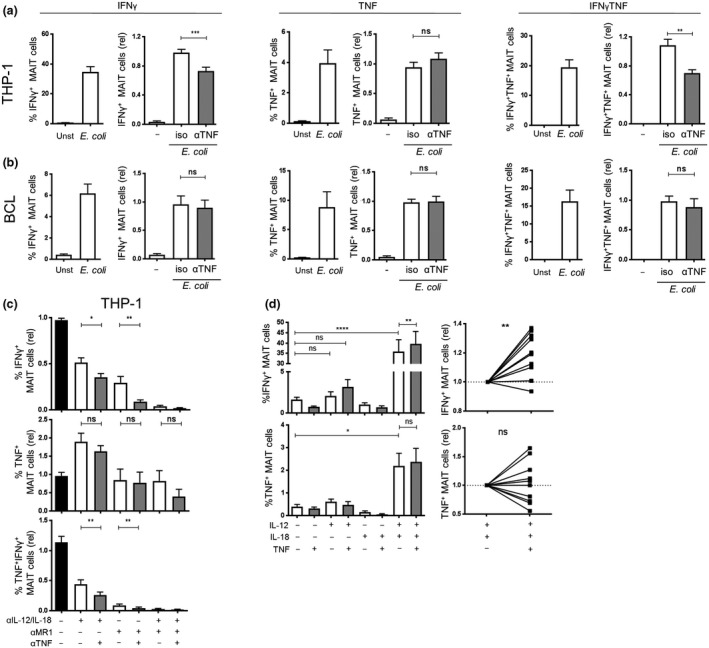
TNF enhances IFNγ expression of MAIT cells by THP‐1 cells but not by BCLs. Enriched CD8 T cells were co‐cultured for 20 h with THP‐1 cells **(a)** or BCLs **(b)** in the absence or presence of *E. coli* (20 BpC) and an isotype antibody or a TNF‐neutralizing antibody. MAIT cells were analyzed for the expression of IFNγ and TNF. Each graph on the left shows the absolute percentage of the indicated cytokine expressing MAIT cells, and each graph on the right shows the relative percentage of the indicated cytokine expressing MAIT cells after stimulation in the presence of an isotype control antibody or anti‐TNF antibody. **(c)** Enriched CD8 T cells were co‐cultured for 20 h with THP‐1 cells and *E. coli* (20 BpC), and the indicated combinations of blocking antibodies for MR1, IL‐12/IL‐18 or TNF. The percentages of MAIT cells expressing IFNγ or TNF are shown. **(d)** Enriched CD8 T cells were stimulated for 20 h with indicated combinations of recombinant IL‐12, IL‐18 and TNF. The graphs show the percentage (left) of MAIT cells expressing IFNγ or TNF. Right graphs show the effect of TNF on IFNγ or TNF expression of MAIT cells relative to IL‐12/IL‐18 treated cells. Data are shown as mean ± s.e.m. of 11 donors pooled from three experiments performed (for a and b), seven donors pooled from two experiments performed for **c** or 4–12 donors pooled from one to three experiments performed for **d**. *P < 0.05, **P < 0.01, ***P < 0.001, ****P < 0.0001, ns = not significant, paired *t*‐test **(a**–**c)** or one‐way ANOVA with Sidak's multiple comparisons test **(c)**.

Cytokines like IL‐12 and IL‐18 have been reported to play an important role in MAIT cell activation^13^; however, TNF has not been studied in this context yet. Thus, we next investigated the contribution of THP‐1 cell‐secreted TNF to the activation of MAIT cells. In order to study this, we co‐cultured THP‐1 cells or BCLs with CD8 cells and *E. coli* in the presence or absence of a TNF‐neutralizing antibody. TNF neutralization resulted in an approx. 20% reduction in IFNγ^+^ and IFNγ^+^TNF^+^ MAIT cells when co‐cultured with THP‐1 cells, whereas no change in the amount of TNF^+^ MAIT cells was observed (Figure 3a). Furthermore, BCL‐induced MAIT cell cytokine production was not altered by TNF neutralization (Figure 3b), consistent with the finding that BCLs did not produce TNF (Figure 2b). Next, we questioned whether TNF neutralization in combination with MR1 or IL‐12/IL‐18 blocking could further inhibit cytokine production by MAIT cells. Supplementing MR1 or IL‐12/IL‐18 blockade with TNF neutralization resulted in a further significant reduction in the frequencies of IFNγ^+^ and IFNγ^+^TNF^+^ MAIT cells (Figure 3c). Moreover, IFNγ^+^ and IFNγ^+^TNF^+^ MAIT cells are essentially absent if MR1, IL‐12/IL‐18 and TNF were neutralized together. In contrast, we could not observe significant changes in the number of TNF^+^ MAIT cells when MR1 or IL‐12/IL‐18 blockade was supplemented with an anti‐TNF antibody (Figure 3c). Finally, we examined the direct effect of TNF on MAIT cells by stimulating CD8 T cells with recombinant TNF in combination with IL‐12 and IL‐18. Consistent with previously reported data,^13^ IL‐12/IL‐18 stimulation induced IFNγ expression in about 35% of the MAIT cells and TNF expression in about 2% of the MAIT cells. Supplementing IL‐12/IL‐18 with TNF further increased IFNγ^+^ MAIT cells by up to 40%, whereas TNF expression remained similar (Figure 3d). Thus, IFNγ induction in MAIT cells was enhanced by TNF.

### IgG, but not complement, enhances MAIT cell activation in the presence of normal human serum

**Figure 4:**
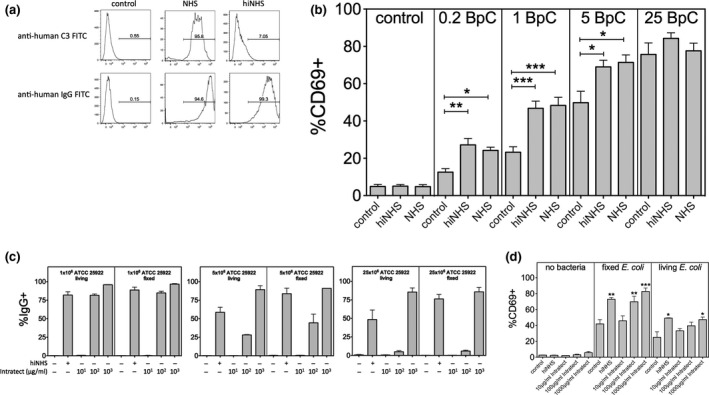
IgG enhances MAIT cell activation in the presence of normal human serum. **(a)** Deposition of C3 and IgG molecules on formaldehyde‐fixed *E. coli* in the presence of normal human serum (NHS) or heat‐inactivated normal human serum (hiNHS). **(b)** Activation of MAIT cells by different amounts (bacteria per cell, BpC) of formaldehyde‐fixed *E. coli* (ATCC2592) in the presence or absence of NHS or hiNHS. Data are shown as mean ± s.e.m. of nine independent experiments. **(c)** Binding of IgG molecules to different amounts (1 × 10^6^, 5 × 10^6^ or 25 × 10^6^) to either formaldehyde fixed or living *E. coli* (strain ATCC25922) in the presence or absence of hiNHS or different concentrations of purified IgG (Intratect). Data are shown as mean ± s.e.m. of two independent experiments. **(d)** Activation of MAIT cells by either formaldehyde‐fixed or living *E. coli* in the presence or absence of hiNHS or different concentrations of purified human IgG (Intratect). Data are shown as mean ± s.e.m. of four independent experiments.

In subsequent experiments using human serum, we adapted our experimental system to a more physiological setting. In human serum, bacteria can interact with pathogen‐specific IgG antibodies resulting in immune‐complex formation and complement activation. In initial experiments, we tested whether formaldehyde‐fixed *E. coli* could be opsonized with complement and IgG molecules in the presence of normal human serum (NHS). We found that both complement C3 and IgG molecules deposited on the surface of *E. coli* after incubation in NHS (Figure 4a: NHS). Heat‐inactivation of NHS (hiNHS), as expected, abrogated the accumulation of C3 on the bacterial surface but did not affect the deposition of IgG antibodies (Figure 4a: hiNHS). Next, we explored the effect of C3‐ and IgG‐opsonization of *E. coli* on MAIT cell activation in PBMCs. We incubated different amounts of formaldehyde‐fixed bacteria with PBMCs in the presence or absence of active NHS or hiNHS. MAIT cells were defined in cultures as CD161^++^TCRVα7.2^+^CD8^+^CD3^+^ cells (Figure 1a). In accordance with previous studies, *E. coli* induced the activation of MAIT cells as determined by the expression of CD69, as a general activation marker for T cells, and the level of MAIT cell activation correlated with the number of bacteria used (Figure 4b). The presence of NHS in the cultures of PBMCs with *E. coli* significantly enhanced the frequency of CD69‐positive, activated MAIT cells when compared to controls without NHS (Figure 4b: control *versus* NHS). Without NHS about five times more bacteria were necessary to achieve the same level of activated MAIT cells compared to that found in the presence of NHS. Surprisingly, cultures with hiNHS showed similar enhancement of MAIT cell activation compared to NHS (Figure 4b: NHS *versus* hiNHS) suggesting that IgG‐ rather than C3‐opsonization of *E. coli* triggers MAIT cell activation. We confirmed these findings using primary monocytes and isolated CD8 T cell in the co‐cultures (Supplementary figure 1a). To accurately state that complement‐opsonization does not enhance MAIT cell activation, we used C5‐deficient serum to avoid potential complement‐mediated lysis and loss of bacteria. Again, cultures with hiNHS and C5‐deficient hiNHS resulted in enhanced MAIT cell activation, but C5‐deficient NHS did not show any further increase in the frequency of activated MAIT cells (Supplementary figure 1b).

To further investigate the effect of IgG‐opsonization on MAIT cell activation, we used a purified IgG pool (Intratect) and confirmed a concentration‐dependent binding of IgG‐molecules to the surface of either formaldehyde‐fixed or living *E. coli* (Figure 4c). More importantly, similar to hiNHS, Intratect induced a significant and dose‐dependent enhancement of MAIT cell activation if PBMCs were co‐cultured with living or formaldehyde‐fixed bacteria (Figure 4d). Together, these data demonstrate that specifically IgG opsonization of *E. coli* can enhance MAIT cell activation.

### IgG‐deposition on *E. coli* enhances the association of bacteria with monocytes, B cells and granulocytes

**Figure 5:**
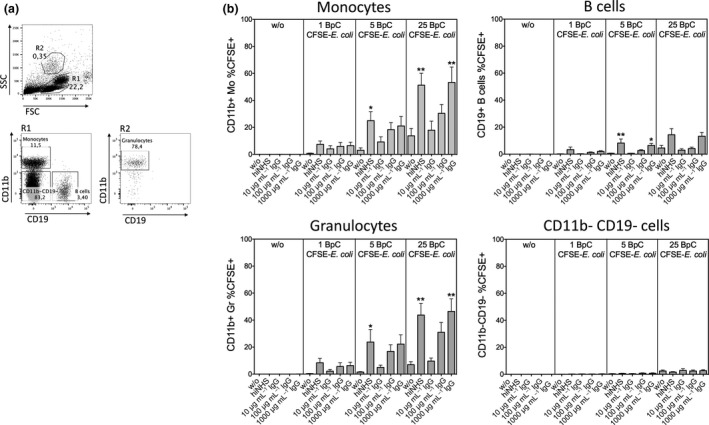
Association of *E. coli* with monocytes, B cells and neutrophils is enhanced in the presence of both hiNHS and purified IgG. **(a)** Gating strategy to define different cell populations in PBMC. In SSC ^low^ gate (R1) CD11b^+^ cells represent monocytes and CD19^+^ cells are B cells. Granulocytes were defined in SSC ^high^ gate (R2) as CD11b^+^ cells. **(b)** Association of different amounts (1, 5 and 25 BpC) of formaldehyde‐fixed, CFSE‐labeled *E. coli* with SSC ^low^ CD11b^+^ monocytes, CD19^+^ B cells, SSC ^high^ CD11b^+^ granulocytes and CD11b^‐^/CD19^−^ cells in the presence or absence of hiNHS or different concentrations of purified human IgG (Intratect). Data are shown as mean ± s.e.m. of seven donors from independent experiments.

Next, we investigated whether the enhanced MAIT cell activation by IgG‐opsonization was due to a better association of *E. coli* with different cell populations relevant for MAIT cell activation in PBMCs. We took advantage of previously described protocols^20^ used to measure bacterial uptake of FITC‐ or CFSE‐labeled bacteria (Supplementary figure 2a). Using CFSE‐labeled *E. coli* we found that the presence of hiNHS or Intratect increased the association of bacteria with PBMCs (Supplementary figure 2b). To further investigate the association of *E. coli* with different cell populations within PBMCs, we defined lymphocytes by gating for FSC^low^CD14^neg^ cells, followed by gating for CD3^neg^CD19^neg^ cells, CD3^neg^CD19^pos^ (B) cells, and CD3^pos^CD19^neg^ (T) cells. Monocytes were defined as FSC^mid^CD14^pos^ cells and neutrophils were characterized as FSC^high^CD14^pos^CD15^pos^ cells (Supplementary figure 2c). We showed that within PBMCs, FITC‐labeled *E. coli* mainly associated with monocytes, B cells and neutrophil granulocytes (Supplementary figure 2c). In subsequent experiments, we assessed whether IgG‐opsonization enhanced bacterial association with monocytes, B cells and granulocytes within PBMCs. IgG and hiNHS significantly enhanced the binding of CSFE‐labeled *E. coli* to CD11b^pos^ monocytes, SSC^high^CD11b^pos^ granulocytes and, to a lesser extent, to CD19^pos^ B cells (Figure 5a, b). In contrast, we found only a marginal interaction of *E. coli* with any other cell type (CD11b^neg^CD19^neg^) present in PBMCs, which was not influenced by IgG‐opsonization (Figure 5b). In summary, IgG‐opsonization of *E. coli* enhances association with APCs .

### IgG‐opsonization of *E. coli* enhances MAIT cell activation by THP‐1 cells but not by B cells

As IgG‐opsonization of *E. coli* elevated the binding or phagocytosis of bacteria to both monocytes and B cells, which can both act as APCs and express MR1,^5^, ^21^ we next questioned whether opsonization of *E. coli* with human IgG serum could enhance MAIT cell activation specifically via these cells. Therefore, we preincubated BCLs or THP‐1 cells with 0.5 BpC non‐opsonized or IgG‐opsonized *E. coli* for 5 or 24 h. Cells were extensively washed to remove non‐phagocytosed and non‐attached bacteria and co‐cultured for 20 h with isolated CD8 T cells. To investigate MAIT cell activation we first examined CD69 expression. Upregulation of CD69 on MAIT cells was clearly detectable after co‐culturing CD8 T cells with *E. coli*‐preincubated THP‐1 cells and was most pronounced after 24 h of preincubation. In contrast, BCLs preincubated with *E. coli* induced only a slight increase in CD69 expression on MAIT cells independent of the time of preincubation (Supplementary figure 3). IgG‐opsonization of *E. coli* further triggered THP‐1‐induced CD69 expression on MAIT cells. The level of CD69 expression on MAIT cells induced by *E. coli*‐IgG preincubated for 5 h with THP‐1 reached the level of MAIT cell activation achieved with non‐opsonized *E. coli* after 24 h (Supplementary figure 3). In contrast, preincubation of BCLs with *E. coli*‐IgG did not enhance MAIT cell activation (Supplementary figure 3).

**Figure 6:**
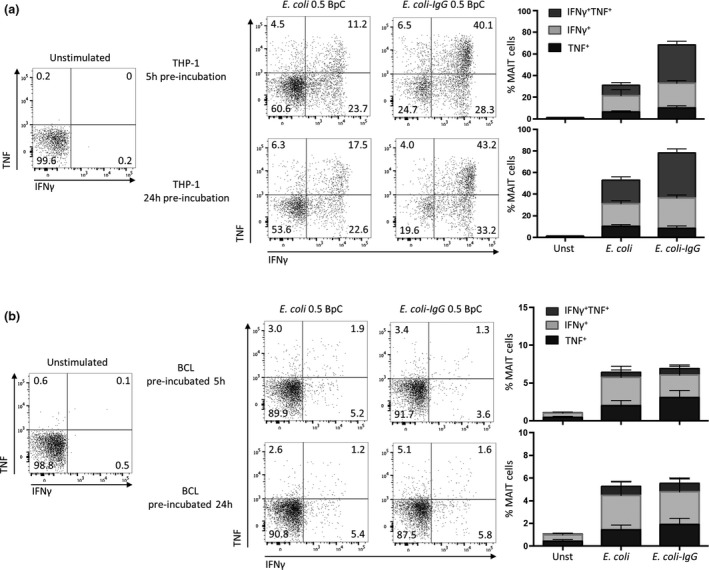
IgG‐opsonization of *E. coli* enhances cytokine production of MAIT cells by THP‐1 cells, but not by BCLs. THP‐1 cells **(a)** or BCLs **(b)** were preincubated for 5 or 24 h in the absence or presence of IgG‐opsonized *E. coli* (*E. coli‐*IgG) or *E. coli*. Enriched CD8 T cells were co‐cultured for 20 h with preincubated THP‐1 cells or BCLs. MAIT cells, gated for as in Figure 1a, were analyzed for the expression of IFNγ or TNF. The graphs on the right show the percentages of MAIT cells expressing IFNγ or TNF. Data are representative of seven (THP‐1) or eight (BCLs) donors or shown as mean ± s.e.m. of seven (THP‐1) or eight (BCLs) donors pooled from two experiments.

Next, we studied the effect of bacterial IgG‐opsonization on the THP‐1‐ and BCL‐induced cytokine responses in MAIT cells by analyzing the expression of IFNγ and TNF. THP‐1 cells induced a higher percentage of cytokine‐producing MAIT cells when *E. coli*‐IgG was used compared to non‐opsonized *E. coli,* independent of the time of preincubation (Figure 6a). This increase was mainly related to an increase in the frequency of IFNγ^+^TNF^+^ MAIT cells, suggesting that THP‐1 cells that were exposed to *E. coli*‐IgG instruct MAIT cells to produce both cytokines. In contrast, IgG‐opsonization did not alter BCL‐induced cytokine expression in MAIT cells, regardless of the duration of preincubation (Figure 6b). Altogether, these data suggest that IgG‐opsonization of *E. coli* can enhance MAIT cell activation and cytokine expression induced by THP‐1 cells, but not by BCLs.

### TNF production increases in THP‐1 cells, but not in B cells in response to IgG‐opsonized *E. coli*

**Figure 7:**
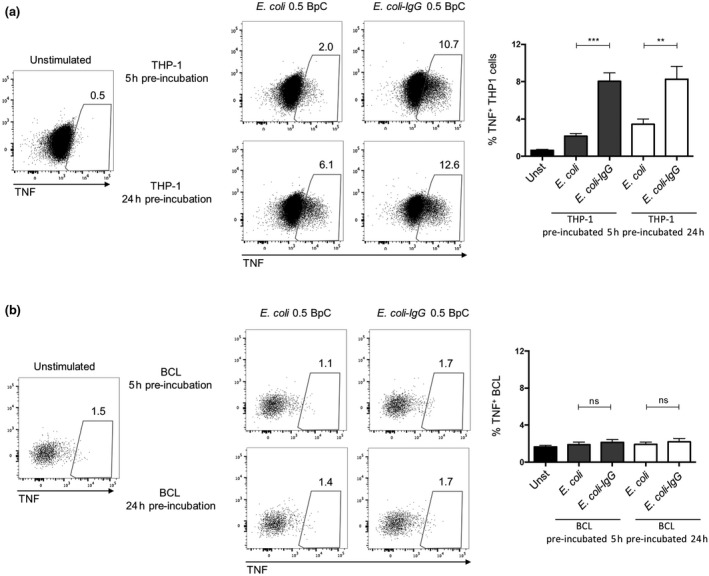
IgG‐opsonization of *E. coli* increases TNF expression in THP‐1 cells, but not in BCLs. THP‐1 cells **(a)** or BCLs **(b)** were preincubated for 5 or 24 h in the absence or presence of IgG‐opsonized *E. coli* (*E. coli‐IgG*) or *E. coli* (0.5 BpC). Enriched CD8 T cells were co‐cultured for 20 h with preincubated THP‐1 cells or BCLs. THP‐1 cells or BCLs, gated for as CD8, were analyzed for the expression of TNF. **P < 0.01, ***P < 0.001, ns = not significant, one‐way ANOVA with Sidak's multiple comparisons test.

Monocytes, once activated by bacterial stimuli, produce cytokines like TNF, which is further triggered upon cross‐linking of FcγRs.^19^, ^22^ We showed above that THP‐1 cells, but not BCLs, respond to *E. coli* by TNF expression in MAIT cell co‐cultures (Figure 2). To expand this observation, we next analyzed TNF production by both THP‐1 cells and BCLs in response to IgG‐opsonized or non‐opsonized *E. coli*. THP‐1 or BCLs were preincubated for 5 or 24 h with 0.5 BpC and subsequently co‐cultured with CD8 T cells. According to our data shown in Figure 2a, b, non‐opsonized bacteria induced TNF production by THP‐1 cells, whereas BCLs did not release substantial amount of TNF upon bacterial stimuli (Figure 7a, b). Using 20 BpC in co‐cultures induced TNF production in about 4–20% of THP‐1 cells, whereas TNF expression by BCLs was seen in only a small fraction of cells (up to 1%). We also investigated the effect of IgG‐opsonization on TNF production by THP‐1 cells and BCLs. IgG‐opsonized *E. coli* could not enhance TNF production by BCLs (Figure 7b), whereas THP‐1 cells displayed a two‐ to fourfold increase in TNF production depending on preincubation times (Figure 7a). In subsequent experiments, we further analyzed bacteria‐induced TNF expression of primary monocytes and THP‐1 cells. Cells were cultivated with *E. coli* or IgG‐opsonized *E. coli* and cytokine release was blocked at different time points by GolgiPlug (BD Pharmingen, San Jose, CA, USA). Using such experimental settings, we found a prolonged production of TNF from monocytes if cells were incubated with IgG‐opsonized *E. coli* (Supplementary figure 4a). Remarkably, unlike primary monocytes which produce TNF and IL‐12 after bacterial stimulation, THP‐1 cells solely secreted TNF, but no detectable IL‐12 in response to *E. coli* (Supplementary figure 4b, c). Thus, IgG‐opsonization of *E. coli* enhances TNF expression in THP‐1 cells and monocytes, but not in BCLs.

### TNF is involved in IgG‐mediated enhancement of MAIT cell activation

**Figure 8:**
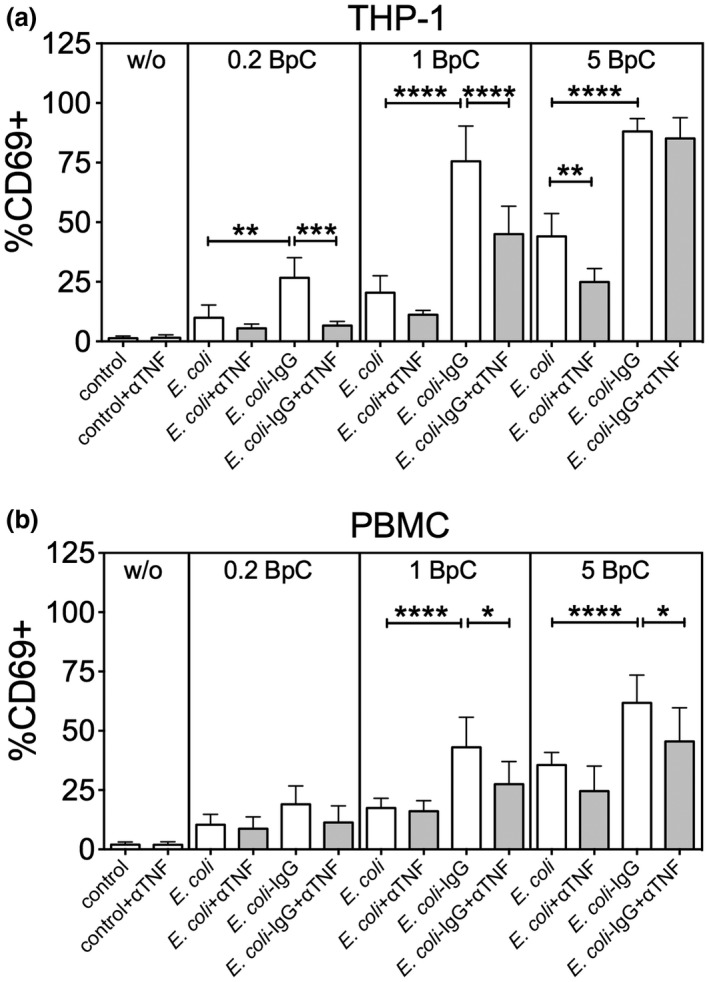
Blocking TNF reduces MAIT cell activation in the presence of both *E. coli* and IgG‐opsonized *E*. *coli*. THP‐1 cells together with isolated CD8 T cells **(a)** or PBMCs **(b)** were co‐cultured with different amounts (0.2, 1 or 5 BpC) non‐opsonized or IgG‐opsonized *E. coli* in the presence or absence of a TNF‐neutralizing antibody (aTNF). Data are shown as mean ± s.e.m. of five (for THP‐1 cells) or seven (for PBMCs) independent experiments.

Finally, we investigated the contribution of TNF in IgG‐mediated enhancement of MAIT cell activation. Again, we performed co‐culture experiments with non‐opsonized or IgG‐opsonized *E. coli* using THP‐1 cells together with isolated CD8 T cells (Figure 8a) or PBMCs (Figure 8b) in the presence or absence of a TNF‐neutralizing antibody. Consistent with our data above, TNF neutralization significantly reduced MAIT cell activation by THP‐1 cells when 5 BpC non‐opsonized *E. coli* was used (Figure 8a). More importantly, IgG‐mediated enhancement of MAIT cell activation by THP‐1 was significantly reduced upon TNF neutralization particularly if suboptimal amounts of bacteria were used (Figure 8a). In a more physiological setting, using PBMCs instead of THP‐1 cells, we found a similar reduction in IgG‐mediated enhancement of MAIT cell activation in the presence of TNF‐neutralizing antibodies (Figure 8b).

## Discussion

Several studies emphasize an important role of MAIT cells in antimicrobial immunity.^5^, ^6^, ^8^ A number of critical aspects in MAIT cell activation have already been defined upon bacterial stimulation, but the regulation of MAIT cell functions has not been fully elucidated. Here we showed a differential activation of MAIT cells by monocytes and B cells in response to *E. coli*. Furthermore, we demonstrated for the first time that opsonization of bacteria with IgG, but not with C3, supports the activation of MAIT cells by monocytes. IgG‐mediated enhancement of MAIT cell activation by monocytes was at least partially dependent on the increased production of TNF, which acted in concert with MR1 and other cytokines to drive MAIT cell activation.

Both bacterially exposed THP‐1 cells and BCLs are regularly used as APCs to study the activation of MAIT cells.^5^, ^13^, ^18^, ^23^ We confirmed these findings but we found differences in the cytokine profile of MAIT cells induced by THP‐1 cells and BCLs in response to *E. coli*. THP‐1 cells are generally more potent in the induction of cytokine production by MAIT cells, generating mainly IFNγ and IFNγTNF but few TNF‐producing MAIT cells. In contrast, BCLs induced fewer cytokine‐producing MAIT cells but interestingly were relatively more potent in the generation of TNF^+^ MAIT cells, although only when high amounts of bacteria (>10 BpC) were used. Nevertheless, increasing the amount of bacteria did not result in more TNF‐producing MAIT cells suggesting that TNF is produced after a certain threshold of stimulation independent of the levels of MR1 and bacterial antigen expression on infected cells. This is in line with a previous study where no correlation was found between MR1 or bacterial antigen expression by BCLs and the proportion of MAIT cells producing cytokines.^18^ However, the induction of IFNγTNF‐secreting MAIT cells by BCLs was dependent on the number of bacteria used. In contrast, we found rather a decrease in IFNγ^+^TNF^+^ MAIT cells in co‐cultures with THP‐1 cells when a higher bacterial load was used, potentially related to the effects of downregulation previously described.^24^ Nevertheless, MAIT cell activation by THP‐1 cells with respect to their CD69 expression was again dependent on the number of bacteria used. This might suggest that the number of bacteria used to induce IFNγ^+^TNF^+^ MAIT cells in THP‐1 co‐cultures is already saturated at 2 BpC. This points to a lower activation threshold of monocytes to induce IFNγ and IFNγTNF‐producing MAIT cells in response to bacteria compared to B cells. Blocking experiments using antibodies for MR1 and IL‐12/IL‐18 showed that IFNγ and IFNγTNF production by MAIT cells is highly dependent on MR1, and also on IL‐12/IL‐18 by both THP‐1 and BCLs. For THP‐1 cells these results are in line with previous findings.^13^ MAIT cell activation by BCLs was described to be dependent on cell–cell contact^18^—additionally our results demonstrate an involvement of IL‐12/IL‐18 signaling in MAIT cell activation by BCLs.

A further difference between THP‐1 cells and B cells in response to bacterial stimulation was found in the TNF production by the APCs themselves. Whereas THP‐1 cells secreted TNF in response to *E. coli*, BCLs did not readily produce it. TNF represents a central cytokine in monocyte and macrophage antimicrobial activity,^19^ and we now showed for the first time that it is also involved in the activation of MAIT cells. During bacterial and viral infections CD8 T cells are, in addition to antigen‐specific stimuli, exposed to an array of cytokines creating a unique inflammatory microenvironment capable of modifying CD8 T‐cell functions.^25^ Bacterial stimulation of human monocytes induces a proinflammatory cytokine signature characterized by the production of IL‐1β, TNF, IL‐6, IL‐8, IL‐12 and IL‐18,^13^, ^26^, ^27^ of which IL‐12 and IL‐18 have already been shown to be involved in MAIT cell activation.^13^ Whereas IL‐12 or IL‐18 on its own stimulates CD8 T cells to produce modest amounts of IFNγ, they act synergistically when combined or used with other cytokines like IL‐7, IL‐15 or IFNα/β, as shown in both human and mouse models.^28^, ^29^, ^30^, ^31^ TNF, a potent mediator of inflammation and antimicrobial immunity, mediates its effects primarily through TNF receptors TNFR1 and TNFR2. TNFR signaling might lead to cell death initiated by TNFR1, but TNFR1‐ and TNFR2‐related signaling can also induce the activation of NF‐κB and MAPK/JNK pathways thereby promoting the production of other inflammatory cytokines.^32^ In line with this, TNF, which alone does not induce a substantial amount of IFNγ, has been demonstrated to be the most potent of the investigated TNF superfamily members acting synergistically with IL‐12 in the induction of IFNγ by CD8 T cells.^29^ In our hands, TNF neutralization alone or in combination with MR1 and IL‐12/IL‐18 blockade in the THP‐1 cell co‐cultures decreased the proportion of IFNγ and IFNγTNF‐producing MAIT cells. TNF signaling in these cells is likely via TNFRII, which we have previously shown to be expressed on MAIT cells and further upregulated after stimulation.^33^

Invading pathogens like bacteria induce both innate and adaptive immune responses. The complement (C) system represents an innate component in the defense against pathogens. C activation by pathogens leads to direct killing by the generation of C5b‐C9 membrane attack complex, triggers inflammatory processes by C’ activation products, and results in C‐opsonization on the activating surfaces. After the induction of adaptive immunity, pathogen‐specific antibodies are generated which also bind to the pathogen surface. Both IgM and IgG antibodies deposited on the pathogen surface further trigger the complement system by initiating the activation of the classical complement pathway. Certain pathogens like *E. coli*, however, have evolved mechanisms to avoid complement attack and therefore are rescued from complement‐mediated lysis.^34^ As a result, pathogens become opsonized with both complement‐fragments and IgG‐molecules under *in vivo* conditions.

We tested this opsonization with *E. coli* and we confirmed that in the presence of active NHS, bacteria became opsonized with both complement and IgG molecules. Heat‐inactivation of serum prevented complement activation and therefore diminished complement deposition, but still allowed the binding of antibodies. *Escherichia coli* binding antibodies were not only detected in NHS but were also found if bacteria were incubated in the presence of a purified IgG pool (Intratect). This is in line with several earlier reports demonstrating the presence of specific‐antibodies in commercial human immunoglobulin preparations against a number of bacteria including *E. coli*,* Klebsiella*, group B *streptococcus* and *P. aeruginosa*.^35^, ^36^, ^37^ According to the findings above, IgG‐opsonization of *E. coli* enhanced the association of bacteria with monocytes, neutrophil granulocytes and B cells. More importantly, opsonization of *E. coli* with IgG, but not with complement, increased the activation and IFNγ/TNF production of MAIT cells by monocytes, but not by B cells.

IgG‐coated particles can bind to FcγR expressed on several immune cells including APCs like monocytes, B cells and dendritic cells. IgG immune‐complexed antigens can be recognized by three different classes of FcγRs: the high‐affinity FcγRI (CD64) and the low‐affinity FcγRII (CD32), FcγRIII (CD16) and the recently identified FcγRIV. Whereas low‐affinity FcγRs can only bind ICs, the high‐affinity FcγRI also binds monomeric IgG molecules.^38^, ^39^ Depending on the presence of immunoreceptor tyrosine‐based activation or inhibitory motifs (ITAMs or ITIMs, respectively) in the intracellular region, FcγRs are divided into two groups: (i) activating FcγRI, FcγRIIa, FcγRIII and FcγRIV or (ii) inhibitory FcγRIIb.^38^, ^39^ THP‐1 cells express FcγRI and FcγRIIa/b, whereas B cells only express inhibitory FcγRIIb.^39^, ^40^ This difference in FcγR pattern between THP‐1 and B cells could explain why IgG‐opsonization of *E. coli* did not influence MAIT cell activation by BCLs.

FcγRs have been shown to trigger binding and subsequent phagocytosis of IgG‐opsonized particles. Moreover, cross‐linking of FcγRs initiates phosphorylation and downstream signaling cascades.^41^ Signaling events related to the engagement of FcγRs lead to the transcriptional activation of several cytokine and chemokine genes, which—depending on the quality of IgG‐FcγRs interactions—directs cells into either a pro‐ or an anti‐inflammatory state.^42^ In murine macrophages, FcγR‐mediated stimulation of MAPK pathways, particularly the activation of p42 MAPK (also known as ERK2) results in TNF synthesis.^43^ Furthermore, antibody‐opsonization of bacteria triggered toll‐like receptor signaling pathways through FcγR resulting in upregulation of various cytokines, including TNF in human dendritic cells.^20^ Thus, IgG‐opsonization of bacteria results in enhanced phagocytosis supporting both MR1‐presentation and an elevated production of TNF by monocytes, which in turn results in more efficient activation of MAIT cells. Since in the absence of IgG about five times more bacteria were necessary to reach the same levels of activated MAIT cells found with IgG, this opsonization might be important to activate MAIT cells sensing limiting amounts of bacteria. Furthermore, the presence of specific antibodies after a first bacterial encounter is expected to accelerate MAIT cell responses upon secondary infections.

Interestingly, during acute *Vibrio cholerae* infection in adult patients, LPS‐specific IgA and IgG responses correlated with both MAIT cell frequencies and activation.^44^ Of note, anti‐TNF therapies are associated with an increased risk of tuberculosis—consistent with the idea that inhibition of MAIT cell activation by anti‐TNF therapies might contribute to this process.^32^, ^45^ Finally, most of the commercial intravenous immunoglobulin G products, used for example in patients with primary antibody deficiency, contain specific antibodies against a range of pathogens.^35^, ^37^, ^46^ Thus, an IgG‐mediated enhancement of MAIT cell function might also be included in the mode of action of intravenous immunoglobulin G therapy. All of these aspects need further investigation to explore the potential *in vivo* relevance of our findings.

## Methods

### Ethics statement

Peripheral blood from anonymized healthy donors was obtained locally from the NHS Blood and Transplant UK and Oxford (approved protocol COREC 04.OXA. 010), or the Central Institute for Blood Transfusion and Immunology, Medical University Hospital Innsbruck, Innsbruck, Austria. The use of anonymized leftover specimens for scientific purposes was approved by the Ethics Committee of the Medical University of Innsbruck. Written informed consent was obtained from all participating blood donors.

### Bacteria and opsonization

*Escherichia coli* (ATCC 25822, NCTC 9001 or DH5α) were fixed in 2% paraformaldehyde for 20 min at room temperature, washed and used for opsonization or co‐cultures. Experiments with non‐fixed bacteria were performed with media supplemented with 1% penicillin/streptomycin. To generate fluorescent‐labeled bacteria, fixed *E. coli* were incubated for 1 h with 10 μg mL^−1^ of FITC isomer I (Sigma‐Aldrich, St Louis, MI, USA) or 10 μm CFSE (Sigma‐Aldrich) at 37°C followed by extensive washing. Formaldehyde‐fixed bacteria were pre‐opsonized in the presence of normal human serum (NHS, from Quidel, San Diego, CA, USA) or NHS heat‐inactivated for 30 min at 56°C (hiNHS) in 1:5 dilution for 1 h at 37°C. Alternatively, bacteria were incubated with different concentrations of purified IgG (Sigma‐Aldrich or Intratect from Biotest, Dreieich, Germany). Opsonized bacteria were washed with PBS, stained with FITC‐labeled anti‐human C3 or anti‐human IgG antibodies (both from DAKO, Santa Clara, CA, USA) and analyzed by flow cytometry.

### Antibodies and flow cytometry

For cell surface staining antibodies/dyes were used as listed in Supplementary table 1. Living cells were detected using viability dye Live/Dead fixable‐near‐IR (Invitrogen, Carlsbad, Germany) or 7‐AAD (BD, Franklin Lakes, NJ, USA). For intracellular cytokine staining cells were permeabilized using a Fix/Perm buffer (BD) and stained with antibodies as listed in Supplementary table 1. Cells were fixed in PBS/1% formaldehyde and measured with a BD FACSCanto II (BD) or a MACSQuant (Miltenyi, Bergisch Gladbach, Germany) flow cytometer. Data were analyzed using FACS Diva (BD) or FlowJo software.

### Human primary cells and cell lines

PBMCs from peripheral blood were isolated by centrifugation on a Pancoll (PanBiotech, Aidenbach, Germany) density gradient. Monocytes were separated from PBMC by adherence on gelatin‐coated petri dishes as described previously.^47^ CD8 T cells were isolated from PBMCs by positive selection with anti‐CD8 microbeads using MS columns (both Miltenyi Biotec, Bergisch Gladbach, Germany) or alternatively by negative selection using BD IMag Human CD8 T Lymphocyte Enrichment Set (BD). Cells were cultured in R10 medium (RPMI 1640 with 10% FCS, 5% l‐glutamine). Epstein–B virus‐transformed BCL were prepared from healthy blood donors obtained as for the PBMC samples. THP‐1 cells were obtained from ATCC. THP‐1 cells or BCLs were cultured in R10 medium supplemented with β‐mercaptoethanol (3.5 nL mL^−1^; Sigma‐Aldrich) and split into a concentration of 0.2 × 10^6^ cells mL^−1^ every 4 days.

### Co‐cultures and stimulations

PBMCs (10^6^) were cultured with fixed *E. coli* using the indicated amounts of BpC in the presence or absence of NHS, hiNHS or different amounts of purified IgG (Intratect) in R10 medium. Alternatively, bacteria pre‐opsonized in the presence of hiNHS or purified IgG (as described above) were used at the indicated BpC numbers. After 20–22 h of culture, MAIT cell activation was analyzed by flow cytometry. Alternatively, THP‐1 cells or BCLs were preincubated with paraformaldehyde‐fixed *E. coli* or IgG‐ *E. coli* in the indicated amounts of BpC for 5 or 24 h at 37°C. After the incubation, enriched CD8 T cells were added at a ratio of 1:2 and co‐cultured for further 20 h at 37°C. Blocking antibodies against IL‐12p40 (5 μg mL^−1^; Clone C.8.6; Biolegend, San Diego, CA, USA) and IL‐18 (5 μg mL^−1^; Clone KU18.81; Biolegend), MR1 (5 μg mL^−1^; Clone 26.5; Biolegend), TNF (5 μg mL^−1^; Miltenyi Biotec), isotype IgG1 (5 μg mL^−1^; Clone 11711; R&D Systems, Minneapolis, MN, USA) or isotype IgG2a (5 μg mL^−1^; Clone 20102; R&D Systems) were added at the beginning of the co‐cultures if indicated. The final 4 h of the co‐cultures were in the presence of Brefeldin A (eBioscience, Waltham, MA, USA). Activation and cytokine production of MAIT cells were analyzed by flow cytometry.

### Statistics

All graphs and statistical analyses were completed using Prism software 7 (GraphPad, San Diego, CA, USA). Statistical significance was determined using paired, one‐tailed *t*‐tests or one‐way ANOVAs with Sidak's or Bonferroni's multiple comparison tests.

## Conflict of Interest

The authors declare no conflict of interest.

## Supporting information


**  **
