# Supplementary material for: Antibody opsonization enhances MAIT cell responsiveness to bacteria via a TNF‐dependent mechanism
Source: Immunol Cell Biol. 2019 Feb 25;97(6):538–51. doi: 10.1111/imcb.12239 (PMC6767153; doi:10.1111/imcb.12239)
Supplement: Supplementary file 1 [file IMCB-97-538-s001.doc]

**SUPPORTING INFORMATION**

**Supplementary Table 1.** Antibodies used for flow-cytometry.

| **antibody** | **clone** | **company** |
| --- | --- | --- |
| CD69-FITC | FN50 | BD (Franklin Lakes) or eBioscience (Waltham) |
| CD161-PerCP/Cy5.5 | HP-3G10 | BioLegend (San Diego) |
| CD161-PerCP/Cy5.5 | REA631 | Miltenyi (Bergisch Gladbach) |
| TCRVα7.2-APC | REA179 | Miltenyi (Bergisch Gladbach) |
| TCRVα7.2-APC | 3C10 | BioLegend (San Diego) |
| CD19-APC | HIB19 | BioLegend (San Diego) |
| CD19-PE | REA675 | Miltenyi (Bergisch Gladbach) |
| CD8-APC/H7 | SK1 | BD (Franklin Lakes) |
| CD8-VioGreen | SK1 | Miltenyi (Bergisch Gladbach) |
| CD8-PE/Vio770 | SK1 | Miltenyi (Bergisch Gladbach) |
| CD8-APC | SK1 | BD (Franklin Lakes) |
| CD3-eFluor450 | OKT3 | eBioscience (Waltham) |
| CD3-PE/Cy7 | UCHT1 | BD (Franklin Lakes) |
| CD3-APC | UCHT1 | BD (Franklin Lakes) |
| CD14-PacificBlue | TuK4 | Invitrogen (Carlsbad) |
| CD4-VioGreen | M-T466 | Miltenyi (Bergisch Gladbach) |
| CD15-PE/Cy7 | HI98 | BD (Franklin Lakes) |
| CD11b-PE/Cy5 | ICRF44 | BD (Franklin Lakes) |
| TNF-PerCP/Cy5.5 | MAb11 | BioLegend (San Diego) |
| IFNγ-PerCP/Cy5.5 | 4S.B3 | BioLegend (San Diego) |
| IFNγ-FITC | 4S.B3 | Miltenyi (Bergisch Gladbach) |

**
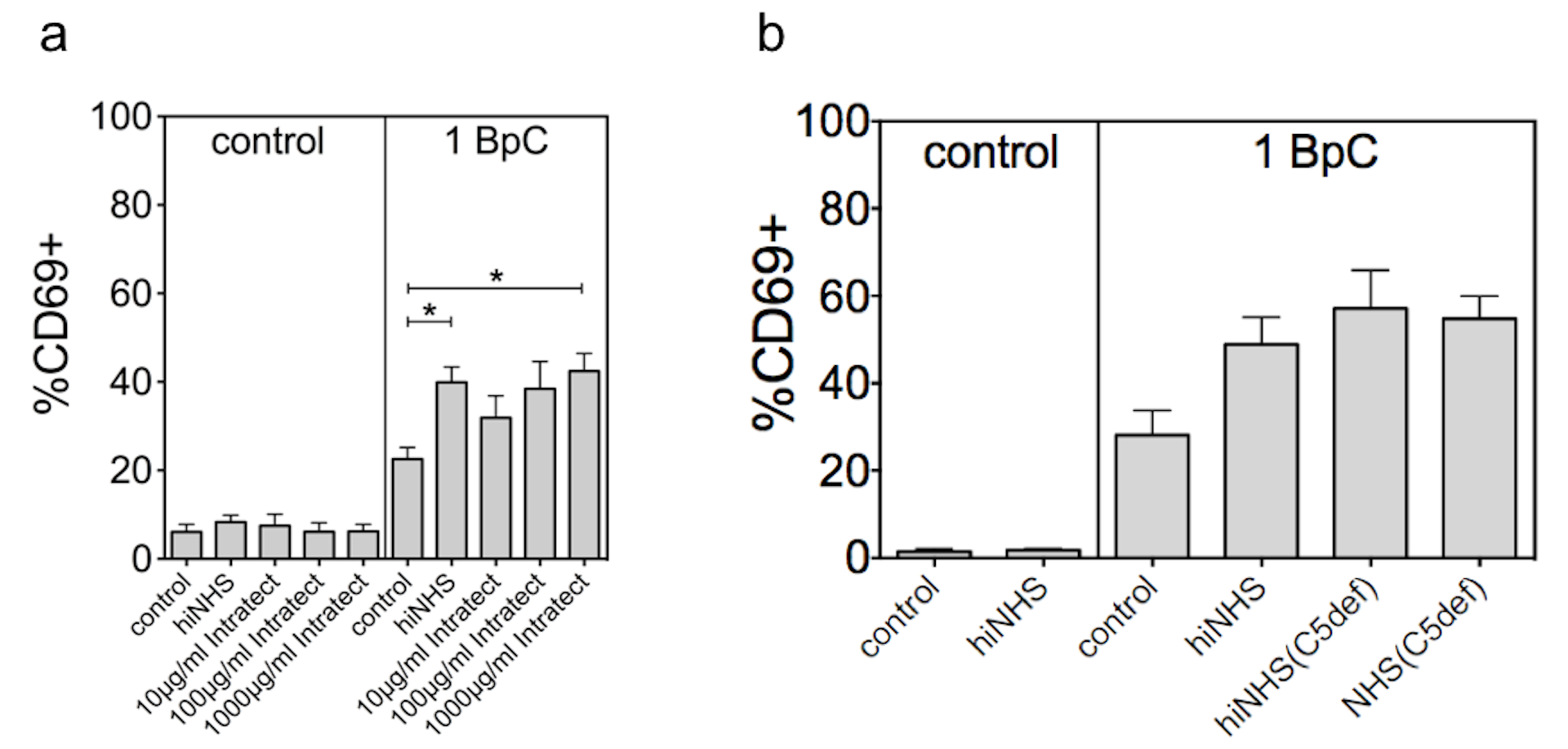
**

**Supplementary Figure 1. MAIT cell activation by IgG-opsonized *E. coli*. (a)** To investigate the role of complement C5-deficient serum (NHSC5def) was used to avoid potential destruction of bacteria by complement. **(b)** IgG-opsonization of *E. coli* enhances MAIT cell activation by primary monocytes isolated from PBMCs.

**
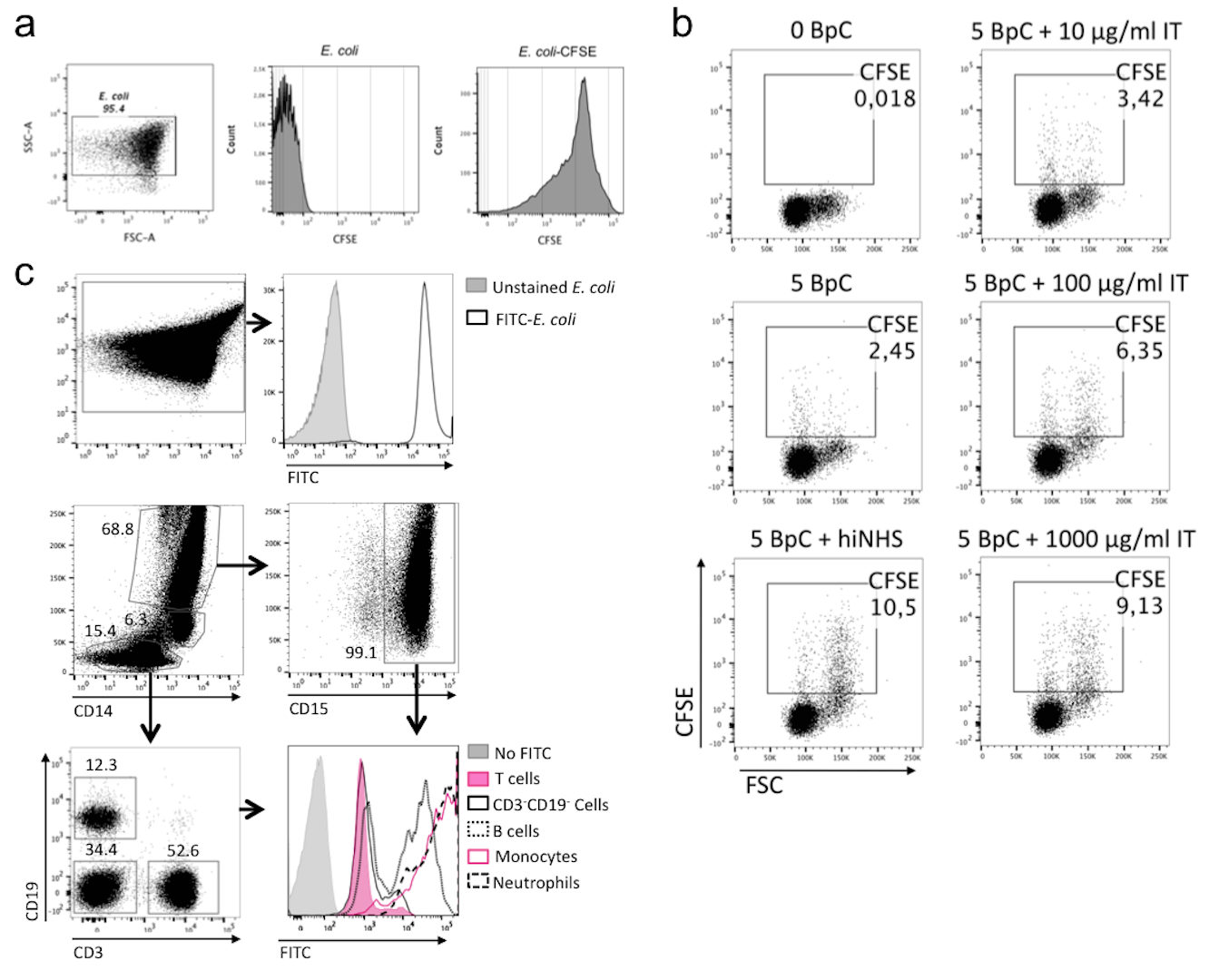
**

**Supplementary Figure 2. *E. coli* associates mainly with monocytes, B cells and neutrophils and association is enhanced by both hiNHS and purified IgG within human PBMCs. (a)** *E. coli* was labelled with CFSE and analyzed by flow cytometry. **(b)** Binding of *E. coli* to human PBMCs. A representative result of PBMCs incubated with or without formaldehyde fixed, CFSE-labeled *E. coli* (5 BpC) in the presence or absence of hiNHS or different concentrations (10, 100 and 1000 μg/ml) of purified human IgG (Intratect, IT). **(c)** Paraformaldehyde-fixed *E. coli* were incubated for 1 hour with FITC and analyzed by flow cytometry. Whole blood was incubated for 4 hours with FITC stained *E. coli* and FITC signal was a measurement for uptake of *E. coli*. Different lymphocytes were defined by gating for FSC^low^CD14^-^ cells, followed by gating for CD19^+^ (B) cells, CD3^+^ (T) cells, and CD3^-^CD19^-^ cells. Monocytes were defined by gating for FSC^mid^CD14^+^ cells and neutrophils were defined by gating for FSC^high^CD14^+^ cells, followed by gating for CD15^+^ cells. Numbers next to gates indicate percentage of cells.

**
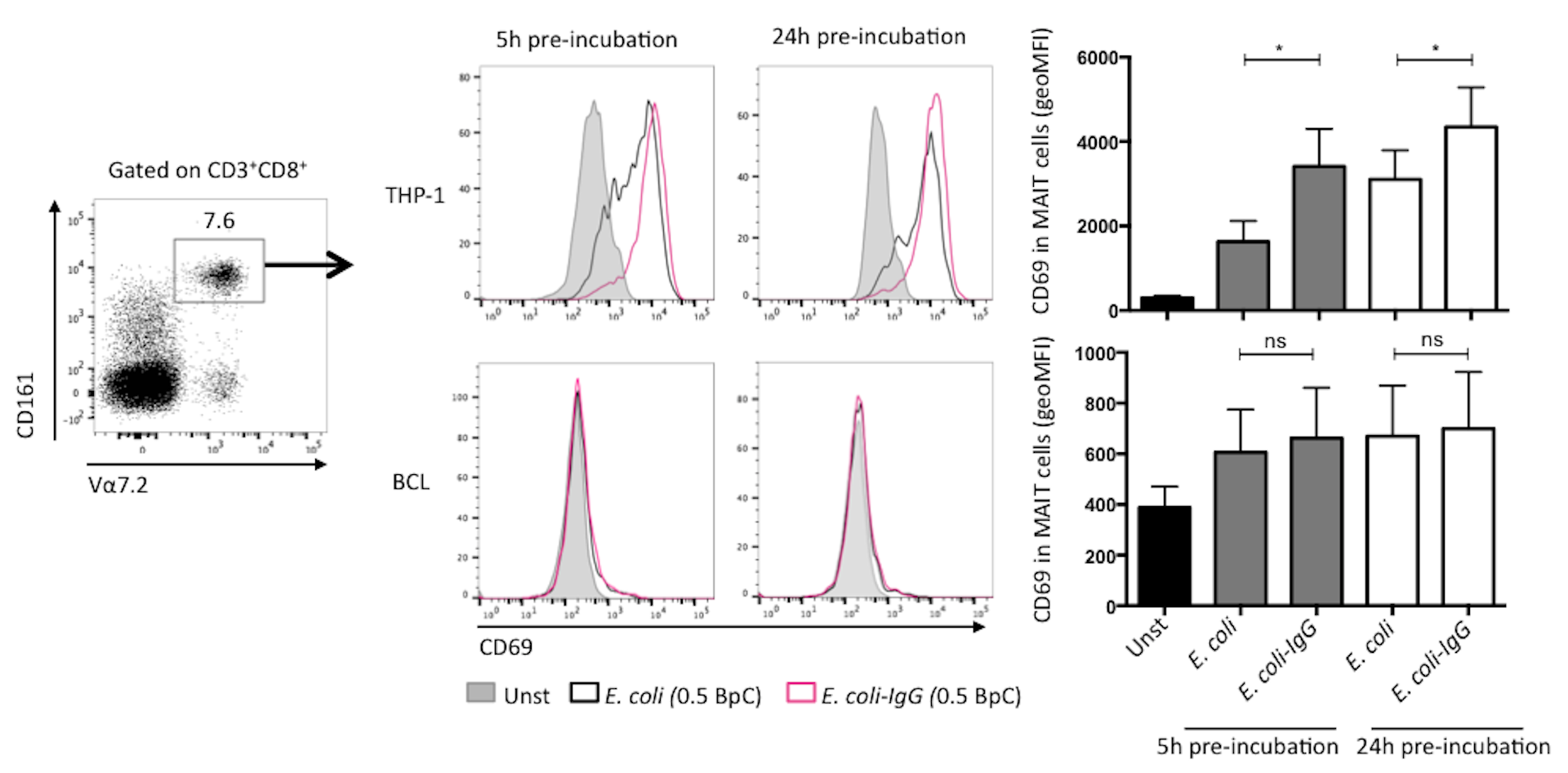
**

**Supplementary Figure 3. IgG-opsonization of *E. coli* enhances MAIT cell activation by THP-1 cells, but not by BCLs.** THP-1 cells or BCLs were pre-incubated for 5 hours or 24 hours in absence or presence of IgG-opsonized *E. coli* (*E. coli-IgG*) or *E. coli* (0.5 BpC). Enriched CD8 T cells were co-cultured for 20 hours with pre-incubated THP-1 cells or BCLs. CD161^++^TCRVα7.2^+^CD3^+^CD8^+^ MAIT cells (left side) were analyzed for the expression of CD69. The graphs on the right show geometric means of CD69 levels on MAIT cells expressing.

**
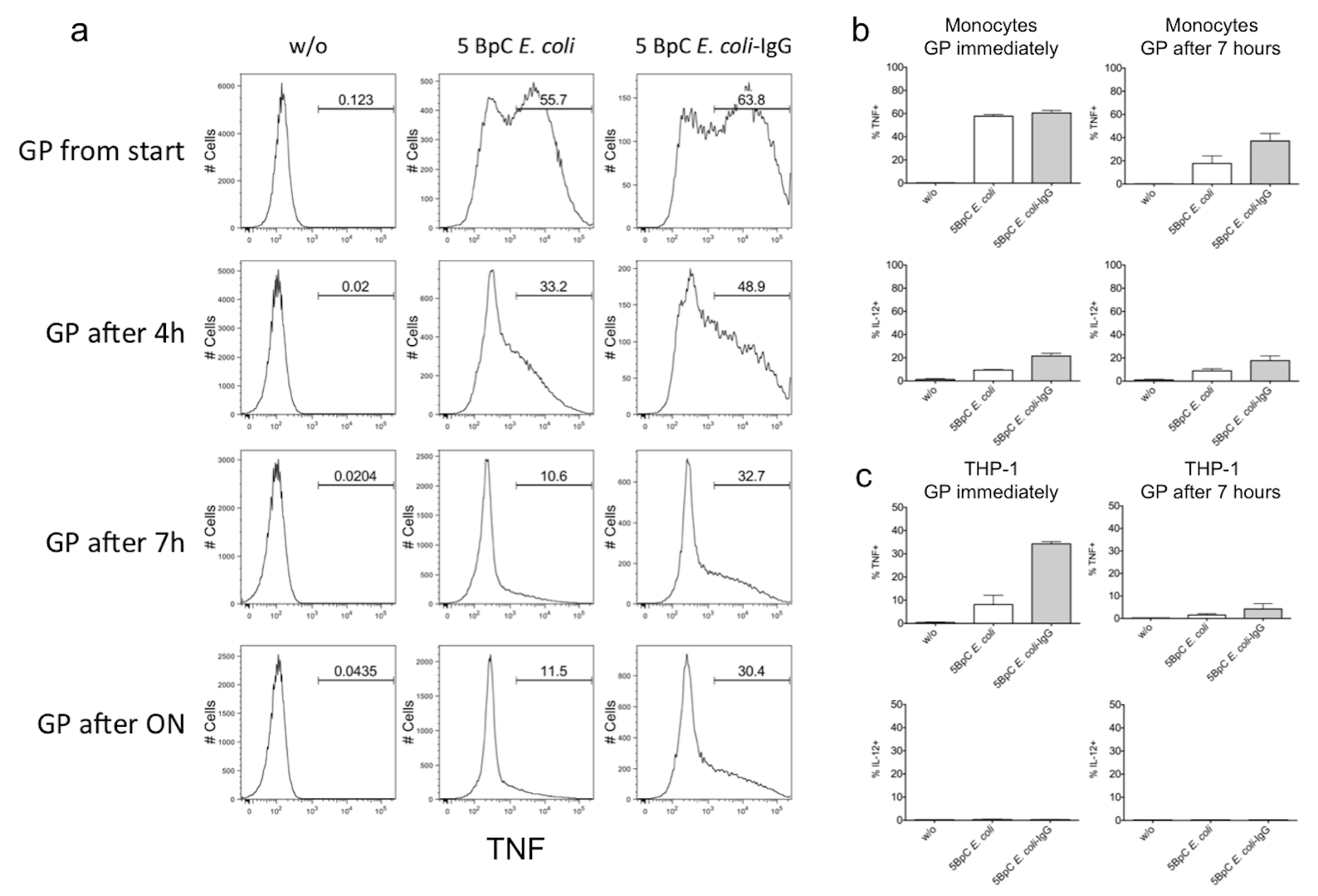
**

**Supplementary Figure 4. IgG-opsonization of *E. coli* prolongs TNFα production of monocytes. (a)** The histograms show the percentages of TNFα production by monocytes cultured with 5 BpC non-opsonized (*E. coli*) or IgG-opsonized (IgG-*E. coli*) formaldehyde-fixed *E. coli*. GolgiPlug™ (GP) was added to the co-culture at indicated time points. Production of TNF and IL-12 from monocytes **(b)** or THP-1 cell **(c)** cultured with 5 BpC non-opsonized (*E. coli*) or IgG-opsonized (IgG-*E. coli*) formaldehyde-fixed *E. coli*. GolgiPlug™ (GP) was added to the co-culture either immediately (GP immediately) or 7 hours after the start of the co-culture (GP after 7 hours).
